# Phytochemical, toxicological and antimicrobial evaluation of lawsonia inermis extracts against clinical isolates of pathogenic bacteria

**DOI:** 10.1186/1476-0711-12-36

**Published:** 2013-12-01

**Authors:** Iram Gull, Maria Sohail, Muhammad Shahbaz Aslam, Muhammad Amin Athar

**Affiliations:** 1 Institute of Biochemistry and Biotechnology, Quid-i-Azam Campus, University of the Punjab, Lahore 54590, Pakistan

**Keywords:** *Lawsonia inermis*, Antimicrobial activity, Time-kill kinetic assay

## Abstract

**Background:**

The emerging resistance of pathogen against the currently available antimicrobial agents demands the search of new antimicrobial agents. The use of medicinal plants as natural substitute is the paramount area of research to overwhelm the drug resistance of infectious agents. Scientists have not made enough effort on the evaluation of safety of medicinal plant yet.

**Methods:**

In the present study antimicrobial activity of *Lawsonia inermis* is investigated against clinical isolates of seven bacteria including four Gram negative (*Escherichia coli*, *Salmonella typhi*, *Klebsiella spp.*, *Shigella sonnei*) and three Gram positive (*Bacillus subtilis, Staphylococcus aureus*, *Staphylococcus epidermidis*) using disc diffusion method. Four types of *Lawsonia inermis* extracts were prepared using methanol, chloroform, acetone and water as extraction solvents, while DMSO (Dimethyl sulfoxide) and water as dissolution solvents. The rate and extent of bacterial killing was estimated by time-kill kinetic assay at 1× MIC of each bacterial isolate. The overall safety of *Lawsonia inermis* extracts was assessed in mice.

**Results:**

*Lawsonia inermis* displayed noteworthy antimicrobial activity against both gram positive and gram negative bacterial strains used in the study. The minimum value of MIC for different bacterial strains ranged from 2.31 mg/ml to 9.27 mg/ml. At 1x MIC of each bacterial isolate, 3log_10_ decrease in CFU was recorded after 6 hours of drug exposure and no growth was observed in almost all tested bacteria after 24 hours of exposure. No sign of toxidrome were observed during *in vivo* toxicity evaluation in mice at 300 mg/kg concentration.

**Conclusion:**

In conclusion, the present study provides the scientific rational for medicinal use of *Lawsonia inermis*. The use of *Lawsonia inermis* extracts is of great significance as substitute antimicrobial agent in therapeutics.

## Introduction

In traditional herbal medicine, plants have been used for many years [1]. Therefore, plants have attained status of natural source of new and potent antimicrobial agents [2]. Medicinal plants are used as ethnomedicine in different countries around the world [3] and are source of natural products providing unlimited opportunity for new drugs because of readily available medicinal diversity [4].

*Lawsonia Inermis* (*L. inermis*) is a scientific name of a tall shrub plant commonly known as Henna or Mehndi [5] or mignonette tree [6] belongs to Kingdom: Plantae, Division: Angiospermae, Class: Dicotyledoneae, Order: Myrtales, Family: Lythraceae, Genus: Lawsonia, Species: L. inermis [7]. Henna is a flowering plant, having a height of 5 meters, natal to subtropical and tropical regions of world including South Asia, Africa, oases of Sahara Dessert and even in northern regions of Australia. Leaves of henna plant are entire, opposite, sub-sessile, oval-shaped and smooth [8]. Leaves have length of 2–3 cm with 1–2 cm width [5]. Henna shrub is highly branched and has greyish-brown barks [9].

Main chemical constituents of henna are Lawsone (2-hydroxynaphthoquinone), mucilage, mannite, gallic acid and tannic acid [10]. Henna is known to be used as a cosmetic agent for dyeing hair, nails and skin [11].

In traditional medicine, henna plant is used to treat many diseases like oedema, bronchitis, menstrual disorder, rheumatism, hemorrhoids and even in jaundice, leprosy, pain, spleen enlargement, dysentery and skin problems [9,12-14]. Henna can also be used as an astringent and antihemorragic agent and is also known for its hypotensive, cardio inhibitory and sedative effects [9]. In addition, henna is reported to show some other properties including hypoglycemic [15], immunostimulant [16], hepatoprotective [17], anti-inflammatory [18], tuberculostatic [19], anti-cancer and antioxidant properties [20].

The present research is designed to determine the antimicrobial activity of leaves of *Lawsonia inermis* available locally in Pakistan against certain pathogenic bacterial strains.

## Materials and methods

### Bacterial cultures

Bacterial cultures used in the present study were clinical isolates collected from the Shaikh Zayed Hospital and Jinnah Hospital, Lahore, Pakistan. The cultures comprise of four Gram negative bacterial isolates namely *Escherichia coli*, *Salmonella typhi*, *Klebsiella spp.*, *Shigella sonnei* and three Gram positive bacterial isolates namely *Bacillus subtilis, Staphylococcus aureus*, *Staphylococcus epidermidis*.

### Maintenance of bacterial cultures

All the bacterial isolates were cultured and maintained in LB (Luria Bertani) medium (1% Tryptone, 1% sodium chloride, 0.5% yeast extract) during all the experiments of the study until mentioned. The bacterial cultures were refreshed fortnightly.

### Plant material

The dried leaves of *Lawsonia inermis* (Henna) used in the present study were purchased from the local market of Lahore, Pakistan.

### Preparation of plant extract

To determine the *in vitro* antimicrobial activity of *Lawsonia inermis*, four different types of extracts including methanol extract, aqueous extract, chloroform extract and acetone extract were prepared.

For the preparation of extracts, dried leaves of Henna were ground to fine powder mechanically in electric grinder. Powdered leaves (10 g) were added in four flasks of 100 ml volume and 50ml of each solvent was added to each flask separately. The flasks were kept in incubator at 37°C overnight with shaking at 180 rpm. The contents of flask were first filtered through four layers of muslin cloth and then through Whatman filter paper. The filtrate was evaporated in rotary evaporator at 50°C. The weight of residues was recorded. DMSO (Dimethyl sulfoxide) was used to dissolve the residues of methanol, acetone and chloroform extracts while aqueous extract residues were dissolved in distilled water at different concentrations. The resulting extracts were stored at 4°C for further use in experiments.

### Inoculum preparation

Before performing antimicrobial activity assay each bacterial strain was refreshed in 5 ml of LB broth (pH 7) separately under sterile conditions. Cultures were incubated in shaking incubator at 160 rpm for 16 hours at 37°C. Each bacterial culture was maintained at the concentration of 10^7^ CFU/ml.

### Antimicrobial sensitivity test using disc diffusion method

The assay of antimicrobial activity of *Lawsonia inermis* extracts was performed by Disc diffusion method [21]. Disc impregnated with DMSO were used as control. The diameter of zones of inhibition formed was measured in mm (millimeters). The test was performed in triplicate with each bacterial strain and mean zone of inhibition was recorded.

### Determination of minimum inhibitory concentration (MIC)

MIC of four different extracts of *Lawsonia inermis* was determined against the test bacterial cultures using the method described by Natta et al. [22] with slight modifications. Briefly, starting from highest concentration of each extract of *Lawsonia inermis* serial dilution were prepared ranging from 544–17 mg/ml for methanol extract, 70–2.18 mg/ml for chloroform extract, 660–20.65 mg/ml for aqueous extract and 74.2-2.31 mg/ml for acetone extract. DMSO was used as diluent for all extract except for aqueous extract where water was used instead of DMSO. Sterile discs were dipped in different dilutions for 1 min and placed on LB agar plates seeded with each bacterial culture separately. The whole experiment was performed under aseptic conditions. Plates were then incubated at 37°C for 16 hrs. The minimum concentration of each extract with clear zone of inhibition was considered as MIC. The zone of inhibition in each case was measured as the diameter of the clear zone and results were recorded. Each experiment was performed in triplicate.

### Phytochemical analysis

The extracts of *Lawsonia inermis* prepared in the present study were screened for phytochemicals including carbohydrates, cardioglycosides, terpenoids, tannins, phenolic compounds, proteins and quinones by phytochemical analysis as below [23].

#### *Carbohydrates*

1 ml of each of four different extracts was taken in test tubes separately and treated with 5 ml of Fehling’s solution (Solution A: 34.6 g of copper (II) sulfate pentahydrate dissolved in 500 ml distilled water, Solution B: 125 g of potassium hydroxide and 173 g of potassium sodium tartrate tetrahydrate dissolved in 500 ml of distilled water, combine solution A and solution B (1:1) just before use). The test tubes were placed in boiling water bath for 5 min. The tubes were observed for appearance of yellow or red color precipitates indicating the presence of reducing sugars.

#### *Cardioglycosides*

5 ml of each of the four extracts was taken in test tubes separately and treated with 2 ml of glacial acetic acid having a drop of ferric chloride solution. 1 ml of the concentrated sulphuric acid was added to each test tube. Test tubes were observed for the appearance of brown coloured ring at the interface indicating the presence of cardioglycosides.

#### *Terpenoids*

5 ml of each of the four extracts was taken in test tubes separately and mixed with 2 ml of chloroform. Concentrated sulphuric acid was added to form a layer. Test tubes were observed for the appearance of reddish brown colour at the interface.

#### *Tannins*

2 ml of each extract of *Lawsonia inermis* was mixed with few drops of 0.1% ferric chloride solution in test tubes separately. Test tubes were observed for the appearance of brownish green colour.

#### *Phenolic compounds*

1 ml of each extract of *Lawsonia inermis* was mixed with 4 drops of ethanol and 3 drops of 0.1% ferric chloride solution in test tube separately. Test tubes were observed for the appearance of red color.

#### *Proteins*

1 ml of each extract was taken in test tubes separately. 2 drops of freshly prepared 0.2% ninhydrin reagent (2.5 g of ninhydrin dissolved in 50 ml n-butyl alcohol on mild heating and stirring and diluted 500 ml with n-butyl alcohol) were added. Test tubes were heated for few minutes. Test tubes were observed for the appearance of blue color.

#### *Quinones*

Few drops of 1 N sodium hydroxide solution were mixed with 1 ml of each extract of *Lawsonia inermis* in test tubes separately. Test tubes were observed for the appearance of red colour indicating the presence of quinones.

### Time-Kill kinetic analysis

The rate of bacterial killing was determined using *Lawsonia inermis* extracts with least MIC value for each bacterial isolate by time-kill kinetic assay as described by Miyasaki et al. [24] with slight modifications. Briefly, overnight bacterial cultures were diluted to the 5 × 10^5^ CFU/mL with LB broth supplemented with 1× MIC of *Lawsonia inermis* extract for each bacterial isolate. The cultures were grown at 37°C with agitation at 160 rpm. The aliquots of cultures were collected at different time intervals (0 hr, 1 hr, 6 hr, 12 hrs, 24 hrs, 36 hrs and 48 hrs), serially diluted in LB broth and plated onto LB agar plates. After incubating the plates at 37°C for 16 hours viable colonies were enumerated. The results were recorded in terms of log_10_ CFU and plotted vs. time for each bacterial isolate.

### Toxicological evaluation of Lawsonia inermis extract

The experiments of acute toxicity in animals were performed in the animal house of Institute of Biochemistry and Biotechnology, University of the Punjab, Lahore, Pakistan after the approval of departmental ethical committee. Each of the two groups comprised of 10 male albino mice (weighing 150-200 g) were used to evaluate the acute toxicity of *Lawsonia inermis* ethanol extract. The animals were housed in cages and served with proper diet according to the international standards. One group was administered with ethanol extract of *Lawsonia inermis* (300 mg/Kg) and other group with equal volume of vehicle (DMSO) daily through subcutaneous route for 2 weeks by subcutaneous injection. The animals were continuously observed for signs of toxidromes such as aggression, sedation, rising fur, increased respiration, altered cardiac rate, excitation, convulsion, stupor, vomiting, etc. or death in first 2 hours and then after 24 hours.

### Statistical analysis

Values are mean of ± standard deviation of three triplicates.

## Results and discussion

**Figure 1:**
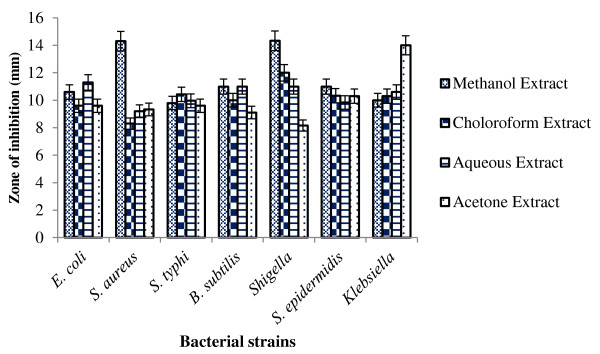
**Zone of inhibition (mm) of *Lawsonia inermis *** **extracts against tested bacterial isolates.**

Dried leaves of *Lawsonia inermis* were used to prepare the extracts as it has been reported that dried preparation have more concentrated active phytochemical compounds than fresh plant material [25]. Four different types of extracts were prepared including methanol extract, chloroform extract, aqueous extract and acetone extract. The results revealed that all extracts exhibited antimicrobial activity against all bacterial strains used in the present study. However bacterial strains showed differential sensitivity for each extract (Figure 1). Antimicrobial activity was not observed with controls (DMSO and water). According to the study of Papageorgiou et al. [26], phytochemical constituents of *Lawsonia inermis* exhibit antimicrobial activity only against gram positive bacteria while ineffective for gram negative bacteria. In our study, it was interested to note that *Lawsonia inermis* had antimicrobial activity against both gram positive (*S. aureus*, *B. subtilis* and *S. epidermidis*) and gram negative (*E. coli*, *S. typhi*, *Klebsiella spp.* and *Shigella*) bacteria. The studies of Bhuwaneshwari et al. [13]; Habbal et al. [27] and Hussain et al. [28] support our findings.

**Figure 2:**
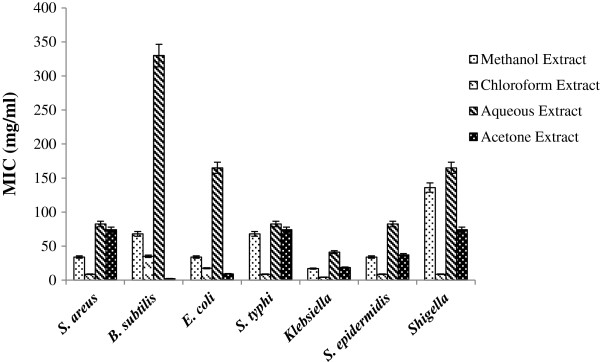
**Minimum inhibitory concentration (MIC) of different extracts of *Lawsonia inermis *** **against tested bacterial isolates.**

The values of MIC for each tested bacterial strain had been shown in Figure 2. From the data in Figure 2, it was illustrated that all of the tested bacterial isolates showed minimum value of MIC for chloroform extract except *E. coli* and *B. subtilis*. The minimum MIC value of *E. coli* (9.27 mg/ml) and *B. subtilis* (2.31 mg/ml) was observed using acetone extract. The MIC values of our study were less than the MIC values reported by Al-kurashy et al. [29]. They found MIC values in the range of 8–64 mg/ml for aqueous extract and 32–64 mg/ml for alcoholic extract of *Lawsonia inermis* against *E. coli*, *S. aureus*, *P. aureginosa* and *E. faecalis*. It was established that chloroform extract of *Lawsonia inermis* was more promising antimicrobial agent for *S. aureus*, *S. epidermidis, S. typhi*, *Klebsiella spp.* and *Shigella* while its acetone extract for *E. coli* and *B. subtilis* at least in *in vitro* antimicrobial assay.

The secondary metabolites mainly attribute the antimicrobial activity of plants [30]. The active constituents of these secondary metabolites include phenolic compounds and tannins [31]. In order to identify the metabolites present in different extracts of *Lawsonia inermis* phytochemical analysis were performed. The data in Table 1 depicted that methanol, acetone and aqueous extracts had cardioglycosides, terpenoids, carbohydrates, phenols, quinones and tannins. While proteins were absent in all three of these extracts. The chloroform extract had cardioglycosides, carbohydrates, phenols, quinones and tannins while proteins and terpenoids were absent. These metabolites solubilized in solvent on the bases for polarity. In most of the plant materials, water soluble components includes starches, tannins, saponins, polypeptides, terpenoids, lectins and different ions [32], while alcoholic extract includes flavonol, alkaloids, tannins, sterols polyphenols etc. [33]. Main chemical constituents of henna include Lawsone (2-hydroxynaphthoquinone), mucilage, mannite, gallic acid and tannic acid [10].

**Table 1:** **Phytochemical analysis of different extracts of *Lawsonia inermis***

**Extracts of *Lawsonia inermis***				
	**Methanol**	**Chloroform**	**Acetone**	**Aqueous**
Cardioglycosides	**+**	**+**	**+**	**+**
Terpenoids	**+**	-	**+**	**+**
Carbohydrates	**+**	**+**	**+**	**+**
Proteins	-	-	-	-
Phenols	**+**	**+**	**+**	**+**
Quinones	**+**	**+**	**+**	**+**
Tannins	**+**	**+**	**+**	**+**

(+ Sign indicating presence of compound; - sign indicating absence of compound).

**Figure 3:**
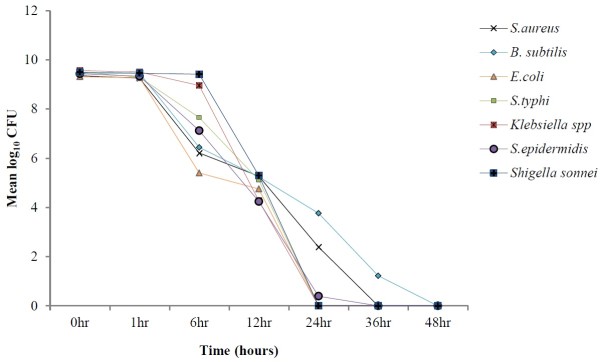
Time-Kill kinetic analysis of tested bacterial isolates.

The rate of bacterial killing after exposure to the 1× MIC of respective extract of *Lawsonia inermis* for each isolate is summarized in Figure 3. The time required to achieve 3log_10_ decrease in CFU is an acceptable index of bactericidal activity from time-kill analysis [34]. The results illustrated that not a single bacterial isolate showed significant bactericidal activity in first hour. Whereas, 3log_10_ reduction in viability of all tested bacterial isolates was observed after 12 hours of exposure. The log_10_ CFU of all bacterial isolates was reduced to zero after 24 hours of exposure except *S. epidermidis* (36 hours) and *B. subtilis* (48 hours).

About 25% of all medicines available in the market have been derived directly or indirectly from plants [35,36]. Herbal medicines are generally believed as safe. However, it is important to evaluate their biological safety before use to avoid fatal consequences [37]. There is no doubt in pharmacological properties of *Lawsonia inermis* but its toxicological assessment is also indispensable. *In vivo* acute toxicity of *Lawsonia inermis* extracts was checked in mice. No mortality was observed during the study. All the signs of toxidrome were negative.

In conclusion, the present study provides the scientific rational for medicinal use of *Lawsonia inermis*. The use of *Lawsonia inermis* extracts is of great significance as substitute antimicrobial agent in therapeutics.

## Competing interests

The authors declare that they have no competing interests.

## Authors’ contributions

All authors equally participated in designing experiments, acquisition, analysis and interpretation of data. Prof. Dr. M. Amin Athar critical revise the manuscript and approved the final version of manuscript. All authors read and approved the final manuscript.
